# Twenty years of home-based palliative care in Malappuram, Kerala, India: a descriptive study of patients and their care-givers

**DOI:** 10.1186/s12904-018-0278-4

**Published:** 2018-02-14

**Authors:** Rekha Rachel Philip, Sairu Philip, Jaya Prasad Tripathy, Abdulla Manima, Emilie Venables

**Affiliations:** 1 Department of Community Medicine, Government T.D Medical College, Alappuzha, Kerala India; 2 International Union Against Tuberculosis and Lung Disease, The Union South East Asia Office, New Delhi, India; 3 Malappuram Initiative in Palliative Care, Malappuram, Kerala India; 4 Medical Department, Médecins Sans Frontières, Brussels, Belgium; 5 Division of Social and Behavioural Sciences, School of Public Health and Family Medicine, University of Cape Town, Cape Town, South Africa

**Keywords:** Home-based care, Palliative care, Community-based care, Care-givers, Chronic disease, Cancer

## Abstract

**Background:**

The well lauded community-based palliative care programme of Kerala, India provides medical and social support, through home-based care, for patients with terminal illness and diseases requiring long-term support. There is, however, limited information on patient characteristics, caregivers and programme performance. This study was carried out to describe: i) the patients enrolled in the programme from 1996 to 2016 and their diagnosis, and ii) the care-giver characteristics and palliative care support from nurses and doctors in a cohort of patients registered during 2013–2015.

**Methods:**

A descriptive study was conducted in the oldest community-based palliative clinic in Kerala. Data were collected from annual patient registers from 1996 to 2016 and patient case records during the period 2013–2015.

**Results:**

While 91% of the patients registered in the clinic in 1996 had cancer, its relative proportion came down to 32% in 2016 with the inclusion of dementia-related illness (19%) cardiovascular accidents (17%) and severe mental illness (5%).Among patients registered during 2013–15, the median number of home visits from nurses and doctors in 12 months were five and one respectively. In the same cohort, twelve months’ post-enrolment, 56% of patients died, 30% were in continuing in active care and 7% opted out. Those who opted out of care were likely to be aged < 60 years, received one or less visit annually from a doctor or have a serious mental illness. 96% of patients had a care-giver at home, 85% of these care-givers being female.

**Conclusions:**

The changing dynamics over a 20-year period of this palliative care programme in Kerala, India, highlights the need for similar programmes to remain flexible and adapt their services in response to a growing global burden of Non Communicable Diseases. While a high death rate is expected in this population, the high proportion of patients choosing to stay in the programme suggests that home-based care is valued within this particular group. A diverse range of clinical and psycho-social support skills are required to assist families and their caregivers when caring for a cohort such as this one.

## Background

Palliative care is gaining significance due to a global increase in the prevalence of Non-Communicable Diseases (NCDs) and the consequent increase in the numbers of patients requiring long-term care for chronic conditions [1]. In spite of the existence of palliative care services in more than half of the world’s countries, there is a need for major expansion for its global accessibility [2].

The South Asian Region which homes one quarter of the world’s population has 1.33 million new cancer cases every year, of which majority present in the later stages of their disease. This is in addition to the burden of other chronic diseases such as end-stage heart failure, cerebrovascular accidents, renal and respiratory diseases which require specialized palliative care [3–6]. Despite this increase in the chronic disease burden, palliative care provision exists in very few countries in the region [7].

Palliative care has been reported to be cost-effective compared to conventional hospital-based and episodic medical care in providing symptomatic relief and improving quality of life [8]. Palliative care is currently provided as home based care or as acute care in hospital setting or as institutional aged care settings sometimes referred to as hospice care [9]. A systematic review by Finlay et al. demonstrated the effectiveness of palliative care, especially home based care over hospital based care with a definite advantage of multi-disciplinary team approach [10]. Home based palliative care involves doorstep provision of holistic care usually by a multidisciplinary team [11]. Home based palliative care existent in European countries provide different models of care, reflecting complexity and context diversity [12].

In United Kingdom, care homes are important providers of palliative care for older people where care is provided by multiple health care service providers who provide care and treatment for residents in care homes [13]. The home care team of specialist palliative care services in Scotland consists of clinicians and nurses who are jointly appointed to work between the independent hospices, the acute hospital sector and community services to provide integrated care [14].

In United States palliative care services are well established within hospitals and hospice care. Hospital palliative care teams focus on caring for seriously ill patients. However, newer models of palliative care delivering services to patients living in the community have been tried recently. Here, interdisciplinary home care is provided through certified home health agencies or hospices outside the Medicare Hospice Benefit whereas hospital-based teams provide physician home visits [15].

Home-based models of palliative care provide home-based care and support for family-care givers as well. It avoids futile treatments and optimizes use of specialist palliative care expertise. Palliative care adopted in the acute care sector largely consists of specialist consultative services, inpatient palliative units/beds or nurse practitioner models [16, 17]. There has also been an expansion of the traditional focus on specialist palliative care teams caring for people with cancer to include a wide range of life limiting diseases [18, 19]. Thus, an ever increasing need to establish appropriate, locally relevant, feasible and effective palliative care for all, irrespective of diagnosis, place of care or geographical region is the current challenge [20].

The various models of palliative care service in India include inpatient care (in hospices or hospitals); outpatient clinics (in hospitals and other settings); and home care services (run by hospitals, hospices or volunteer networks) [21]. The state of Kerala harbours 90% of the country’s palliative care programmes though it has only 3% of the country’s population. This is characterized by a large number of home care services, which have wider coverage [22]. This home-based provision relies on the strength of family support and the enthusiasm of the volunteers. Informal care by family members is unique to this region compared to the developed world where care giving is usually in the form of paid formal care. [23, 24]. Most families prefer to care for the ill person throughout their illness. Most of the literature on feasibility and acceptability of palliative care in India has been based on the models in Kerala [25]. The home-based care services ensure continuity of care for patients and empower the caregiver in the family by teaching them simple and cost effective methods of caring for the patients. This is important in resource poor settings where access to institutional care is difficult [26].

Kerala pioneered community-based palliative care through a socially innovative approach called the Neighbourhood Network in Palliative Care in an attempt to develop a free of charge, sustainable, community led, service capable of offering comprehensive long-term care and palliative care. The network aimed to empower local communities to look after their chronically ill and dying patients. Funds for running the programme were raised locally by volunteers.

The programme was first piloted in 1996 in Malappuram, a northern district of Kerala, for patients with cancer which was then gradually expanded to include other conditions requiring long-term care such as cerebro-vascular accidents, dementia, paraplegia and psychiatric illness [27]. Volunteers from the local community were trained to identify the psycho-social problems of people with chronic conditions in their area and to intervene effectively with active support from a network of trained professionals [27]. The model was shown to be successful, and inspired the state to implement a palliative care policy to ensure universal coverage of palliative care services in all its local administrative units (~ 1000 in numbers), making it the first state in Asia to develop such a policy [28]. The Policy considers home based care as the corner stone of palliative care services. The Neighbourhood Network in Palliative Care in Kerala as discussed above, has been described as an exemplar model of community-based palliative care for other low-resource countries world- wide [21].

Although various provisions of Kerala model of home-based palliative care have been described, there is limited information on patient characteristics and utilization of palliative care [25]. Earlier studies have reported that around 12–28% of patients chose to opt out of palliative care for various reasons which increases with a decrease in social support from the clinic and the number of home visits by the physician [29, 30]. Understanding the patient characteristics, utilization of home-based palliative care and its predictors can assist service planners in the appropriate allocation of resources and service packaging to meet the complex needs of palliative care patients [24, 30].

Manjeri Pain and Palliative Care Clinic is the oldest community based palliative care clinic in Kerala, and understanding its evolution from 1996 to present may be useful in giving insights about patients and caregivers.

### Objectives

The present study was carried out to: i) describe the demographic characteristics of patients enrolled in the programme from 1996 to 2016 and their diagnosis, ii) describe the professional palliative care support they received and the 12-month care outcomes (in active care/opted out of care/transferred out/died) among patients registered between 2013 and 2015, and iii) determine the factors associated with opting out of palliative care.

## Methods

### Study design

A descriptive study was conducted analyzing data from annual patient registers between 1996 and 2016 at Manjeri Palliative Care Clinic. For the second and third objectives, analysis of secondary data from patient case records among those registered between July 2013 and June 2015 was carried out. Outcome of only two years was taken because the documentation of patient case records became systematic only after 2013.

### Setting

The study was conducted in the town of Manjeri, Kerala, a state in the southernmost part of India. The state of Kerala enjoys the highest literacy rate, highest life expectancy and the highest sex ratio in India [31]. The town is located in the most populous district of Kerala (i.e. Malappuram) which has the lowest Human Development Index of 0.75 in the state, which has an overall index of 0.77 [32].

The first community based palliative care project of Malappuram district was in Manjeri. [33] Based on this model and the Kerala State Palliative care policy, the Panchayats/Municipalities (also known as county) in the district of Malappuram currently has two community based palliative care units, one run by the Community Based Organisations (CBOs) and one run by the Local Self Government Institutions (LSGIs). This study focused on the palliative care services provided by CBOs, which offered home-based care service to patients with a variety of long term illnesses through pain and palliative care clinics run by a team of doctors, nurses and volunteers. Nurses visit patients who require support in their homes, including catheterisation, nasogastric intubation, wound care and counseling support. Home visit by doctors is restricted to those patients who require a doctors’ consultation, as charted by a nurse. Volunteers also visit patients and their families to provide a range of support including emotional and financial [33].

### Study population

The study population comprised of all patients who were newly registered in the unit between January 1st 1996 and 31st December 2016. To assess the palliative care outcomes, we looked at patients newly registered in the unit between July 2013 and June 2015.

### Outcome variables and data sources

The key outcome variable was patient’s continuity/retention in the palliative care programme after one year of their enrolment in the clinic which was expressed as “in active care” where the patient was alive and still receiving home based palliative care from the same unit. Transferred out was defined as relocation to another care facility. Opted out of care was defined as the patient’s decision to stop receiving care from the programme.

Sources of data were annual patient registers and patient case records at the Manjeri Pain and Palliative Care Clinic, Kerala. The annual registers contain the name, age and diagnosis of the patient and these have remained uniform over the last two decades. The patient case records contain baseline as well as follow-up information of the patients registered and are maintained by the caring nurses. These case records have been revised from time to time since 1996 and the latest revision was in 2013 whereby documentation became uniform and systematic.

Other operational definitions: The clinic classified the patients into four categories: Very poor, poor, middle and wealthy based on their household characteristics and ability to purchase healthcare. Very Poor-no earning member in the family; Poor-earning member present, but need support for medicines; Middle class-no support needed for daily living but partial financial assistance is needed for medicines; Wealthy-they don’t need external financial help for supporting healthcare. They receive nursing care from the clinic but drugs are not provided free of cost.

For the purpose of this study, very poor, poor and middle class were clubbed into one category i.e. poor because they had limited means to support their treatment/medicines.

### Statistical analysis

Data were entered using EpiData (version 3.1 for entry) and analyzed using EpiData analysis version 2.2.2.182 (EpiData Association, Odense, Denmark) and STATA Version 12. To ensure data quality, double data entry and validation was done. Categorical variables such as socio-demographic and clinical characteristics and care outcomes were summarized as proportions. To summarise continuous variables including the number of visits, median with Interquartile Range (IQR) was used. Chi square test was done to assess the associations of socio-economic and clinical variables with opting out of care. The strength of association was expressed using relative risk (RR) with 95% Confidence Intervals. All factors with a *p* value < 0.2 in unadjusted analysis were put in a log binomial regression model to find out the independent predictors of “opting out of care” using STATA version 12.

## Results

A total of 5614 new patients were registered in the Manjeri Palliative Care Unit over a 20 year period (1996–2016). The demographic and clinical characteristics are shown in Table 1. Sixty-three percent of the newly registered patients were elderly (> 60 years), with only 1% aged 15 years or under. The most common primary diagnosis at enrolment into the palliative care unit was cancer (61%). There were more men than women enrolled in the programme (57% and 43% respectively). (Table 1).

**Table 1:** Demographic and clinical characteristics of newly registered patients in the Manjeri Palliative Care Unit, Kerala, India during 1996–2016

Characteristics	Number	(%)
Total	5614	(100)
Age		
< 15 years	62	(1)
15–44 years	681	(12)
45–59 years	1321	(24)
60–79 years	2443	(44)
≥ 80 years	777	(14)
Missing	330	(6)
Sex		
Male	3206	(57)
Female	2396	(43)
Missing	12	(< 1)
Disease		
Cancer	3445	(61)
Cerebrovascular accident	612	(11)
Dementia related	419	(6)
Diabetes Mellitus	125	(2)
Chronic kidney disease	114	(2)
Peripheral vascular disease	112	(2)
Coronary heart disease	106	(2)
Fracture	102	(2)
Serious Mental illness	78	(1)
Paraplegia/Quadriplegia	76	(1)
Other	362	(6)
Missing	201	(4)

**Fig. 1:**
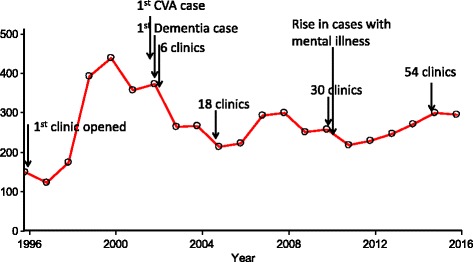
Number of newly registered patients enrolled in the Manjeri Palliative Care Unit, Kerala, India during 1996–2016

**Fig. 2:**
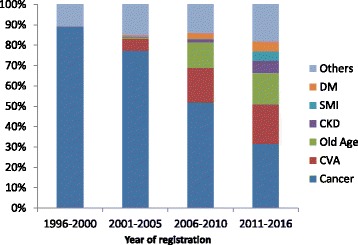
Clinical diagnosis of newly registered patients in the Manjeri Palliative Care Unit, Kerala, India, 1996–2016. CVA = Cerebrovascular Accident; CKD = Chronic Kidney Disease; DM = Diabetes Mellitus; SMI=Serious mental illness; Other category includes Peripheral Occlusive Vascular Disease, Paraplegia, Hypertension, Fractures, Coronary Artery Disease

The number of new patients enrolled annually from 1996 to 2016 is shown in Fig. 1. A rapid increase in the number of patients with CVAs, dementia related conditions, diabetes and serious mental illness is seen up until 2000, when a decline in enrolments is observed thereafter. The diagnosis of patients over these two decades is shown in Fig. 2. While cancer remains the major cause for enrolment, the proportion of patients enrolled due to cerebrovascular accidents (CVA), chronic kidney disease, severe mental illness and dementia related disorders has increased.

Almost all patients enrolled in the programme between 2013 and 2015 (96%) had a care-giver (Table 2). Most of the caregivers (85%) were women. The majority of care-givers (84%) were relatives, with almost one third (31%) being the spouse of the patient. After 12 months in the programme, over half (56%) of the patients enrolled between 2013 and 2015 had died. Just under one third (30%) were still in active care, with 7% having opted out, or chosen to discontinue, in the programme.

**Table 2:** Socio-demographic and clinical characteristics, 12-month care outcomes and palliative care support of patients registered in the Manjeri Palliative Care Unit, Kerala, India, 2013–2015

Characteristics		N	(%)
Total		551	(100)
Age	< 15 years	2	(< 1)
	15–44 years	59	(11)
	45–59 years	82	(15)
	60–79 years	233	(42)
	≥80 years	175	(32)
Sex	Male	265	(48)
	Female	280	(51)
	Missing	6	(1)
Primary diagnosis	Cancer	151	(27)
	Cardiovascular accident	123	(22)
	Dementia related	59	(11)
	Serious Mental Illness	33	(6)
	Fracture	45	(8)
	Chronic kidney disease	34	(7)
	Other	106	(19)
Secondary diagnosis	Dementia related diseases	35	(6)
	Fracture	18	(3)
	Coronary artery disease	17	(3)
	Serious Mental Illness	9	(2)
	Seizure disorder	9	(2)
	Metabolic Encephalopathy	8	(1)
	Others	8	(1)
	None	447	(81)
12-month care outcome^a^	Died	309	(56)
	In active care	164	(30)
	Transferred out of care	38	(7)
	Opted out of care	40	(7)
Care-giver status	Care-giver present	530	(96)
	Care-giver not required	11	(2)
	Living alone	5	(1)
	Missing	5	(1)
Type of care-giver	Spouse	171	(31)
	Daughter in law	139	(25)
	Son/Daughter	107	(20)
	Mother/Father	32	(6)
	Hired care-giver	11	(2)
	Sister-in-law	13	(2)
	Other	73	(13)
	Missing	5	(1)
Median (IQR) number of home visits made by professional care providers 12 months after patient enrollment in the programme^b^			
Doctors		1	(1–2)
Nurses		5	(2–9)

^a^In active care is defined as the patient being alive and still receiving home based palliative care. Transferred out is defined as relocation to another care facility; opted out of care is defined as the patient’s decision to stop receiving care from the programme;

^b^*IQR* Inter Quartile Range

Nurses provided more home visits than doctors, with patients receiving a median of one visit from a doctor and five visits from a nurse in the 12 months following their enrolment in the programme. (Table 2).

The factors significantly associated with opting out of palliative care in unadjusted analysis were patients with age ≤ 60 years, serious mental illness, one or less home visits from doctors and five or less home visits from nurses in their first 12 months of enrolment in the programme. Those patients with CVA were significantly less likely to opt out of care. After log binomial regression, the independent predictors of opting out of care were age ≤ 60 years, having a serious mental illness and receiving one or less visit from doctors. Patients with CVA remained significantly less likely to opt out of care. (Table 3).

**Table 3:** Factors associated with opting out of palliative care among newly registered patients in the Manjeri Palliative Care Unit, Kerala during 2013–2015

Variables	N	Opted out of care N (%)	Unadjusted RR(95%CI)	*p*-value	Adjusted RR(95%CI)	*p*-value
Age group^a^				< 0.001		
≤ 60	165	27(16)	5.1(2.7–9.8)		3.7 (1.9–7.4)	**< 0.001**
> 60 years	375	12(3)	ref		ref	
Sex^a^				0.3		
Male	265	22(8)	1.4 (0.8–2.6)		–	–
Female	280	17(6)	ref		–	–
Socio-economic status^a,b^				0.25		
Poor	260	21(8)	1.9(0.6–5.5)		–	–
Middle class/Wealthy	244	16(7)	ref		–	–
Type of care-giver				0.09		
Spouse	168	13 (8)	0.9(0.5–1.7)		–	–
Other	372	27 (7)	ref		–	–
Primary clinical				< 0.001		
diagnosis						
Cancer	151	11(7)	0.9 (0.5–1.9)		0.7 (0.4–1.4)	0.3
Serious Mental Illness	33	9(27)	3.7 (1.8–7.5)		1.9 (1.3–3.8)	**0.01**
Other	244	18(8)	ref		ref	
CVA	123	2(2)	0.2(0.05–0.9)		0.3 (0.08–0.9)	**0.04**
Number of home visits in first year of enrollment by doctor				0.03		
≤1	293	31 (10)	2.9(1.4–6.1)		2.4 (1.2–6.0)	**0.03**
> 1	247	9(4)	ref		ref	
Number of home visits the first year of enrollment by nurse				0.019		
≤ 5	283	28 (4)	2.1(1.1–4.1)		1.2(0.7–2.7)	0.1
> 5	257	12 (2)	ref		ref	

*CVA* Cardiovascular Accident, *RR* Relative Risk, *CI* Confidence Interval

^a^data missing

^b^This is based on the clinic’s standard assessment of a patient’s socio-economic status depending on whether the breadwinner is ill and the level of economic self sufficiency of the family

^$^Other include children, daughters-in-law, parents, sisters-in-law and hired caregivers

Figures in bold font indicate significant *p* value (< 0.05)

## Discussion

A differing pattern in enrolment of patients into the home care programme was seen from 1996 to 2016. Although cancer remains the most common diagnosis, an increasing proportion of CVAs, dementia related conditions, diabetes and serious mental illness was seen when compared to the initial years of the programme.

Globally, cancer is one of the predominant conditions for which palliative care is offered [2]. A study conducted in Brazil revealed that amongst patients with palliative care needs, non-malignant diseases such as dementia and cerebrovascular diseases were the most common [34]. Cancer formed the predominant group in our study. This was consistent with a report from a similar setting in Kerala where 50% beneficiaries of its home based palliative care were cancer patients [35]. Most of the palliative care services in other parts of the country cater to cancer patients [21, 25]. We observed an increasing proportion of other illness in our study as years progressed which is mainly due to demographic transition fuelling a rapid rise in NCDs and other illnesses related to ageing, meaning that patients requiring care for chronic conditions are no longer only those with cancers [36]. The diversity of patient diagnoses suggests that many people, such as those who are paraplegic or suffering from mental illness, may require medical and psycho-social support for longer periods.

The initial rise in the number of patients registering at the clinic can be linked to the fact that at the time it was the only facility in the area providing palliative care, and it provided for patients not only from the study district of Malappuram but also from adjacent districts. This wide catchment area explains the rise in the number of patients up until 2000, whereas the decline that is observed thereafter is due to the establishment of five other clinics in the district from 2001. This decreasing number of patients with the coming up of new clinics is reported from a similar setting in Kerala (30). By 2008 there were 29 such clinics providing palliative care services. This decline was probably halted by the surge in admissions due to other chronic illness as described above. Other palliative care clinics opened during the 20 year study period, which may also explain some of the changes in enrolment rates.

Not surprisingly, the elderly patients were the predominant beneficiaries of the programme. [29, 35] In the cultural context of Kerala and India, family is the most vital non-formal social security and the most preferred living arrangement. National policy for the elderly upholds the concept of home care services for the aged and outlines institutional care as the last resort [37]. There are very few residential aged care arrangements in the district of Malappuram and this highlights the importance of home based palliative care services for the elderly. The palliative care policy of the state is also in line with the appropriateness of home based service in this context [28].

The majority of the care-givers were females, and were mostly the spouses or daughters-in-law of patients. The predominant number of female care-givers (particularly spouses and daughters-in-law) is also unsurprising in this context, where women traditionally take on the majority of household tasks, and these figures reflect the traditional gender roles within Kerala as a society. This gendered division of care-giving and informal, unpaid labour also exists in many other contexts, where women are sole providers of unskilled and unwaged care for family members with chronic medical conditions or disabilities, including the elderly and those with serious mental illnesses [38–40]. Being a care-giver entails a huge emotional, physical and social burden alongside the physical demands of routine household chores [41]. The stresses placed on care-givers suggest the need for structured palliative care programmes supported by nurses and physicians, that offer psycho-social support to the families of patients in addition to medical care.

A year after enrolment into the programme more than half of the patients died but among those who did not die, most preferred to continue receiving care in the home-based programme. The overall high death rate in our study suggests that palliative care still has an important role to play in giving people, including the elderly, essential pain relief and support towards the end of their lives. The death rate was observed to be higher in this clinic than two similar palliative care clinics (10%–37%) in other districts in Kerala. [29, 30]. The higher death rate probably reflects the active follow up mechanism and hence the enhanced death reporting.

Studies have reported that utilization of palliative care is determined by socio-economic status. Patients with a low socio economic status were more likely to access home based palliative care services and patients with higher socioeconomic status tended to prefer institutional specialty care [30, 42]. We did not find any difference in utilization between patients classified as poor or wealthy. This might reflect the level of acceptability and performance of the palliative care programme in Manjeri as well as the specifics of the context in Kerala.

Patients opt out of palliative care due to two reasons; either when they get cured and no longer require the support of palliative care team or when they prefer other methods of care [30]. The opting out has to be discussed in the context of the service provision ie whether it is provided free or charged. In settings where palliative care is mostly a paid service, socio-economic status and availability of informal care provider determine use of palliative care [42]. In Kerala, home care visits by doctors and nurses, medicines and consumables are provided free of cost to patients [30]. The reason for patients opting out of home based palliative care in another setting in Kerala has been reported to be lack of social support [30]. The rate of opting out from Manjeri clinic was lower compared to another clinic in the neighbouring district. (7% vs 12% in Thrissur) (29). Several other predictors of the use of home-based palliative care service are also reported in literature [30].

We found out that those aged less than 60 years, those with serious mental illness and those who received few doctor visits were more likely to opt out of care.

Those who opt out of care are younger and this is consistent with literature [29]. The elderly was the predominant beneficiary group of this palliative care programme. We speculate that the elderly patients might have more severe disease compared to their younger counterparts, rendering them dependent on palliative care services.

The two morbidities which predicted patient’s decision to continue in care were CVA and mental illness. Patients with CVA continued to stay in palliative care longer, compared to patients with other conditions. Patients with CVA formed the predominant group cared for in a similar setting [29].

We also found that those with Serious Mental Illness (SMI) opted out of palliative care. This is an important finding as this category receives predominantly clinic based care and fewer home visits from doctors and nurses. The service for mentally ill patients is mainly through the weekly psychiatry consultation in the clinic. Patient’s family is reminded of the follow up through telephone by the palliative care nurse. The reason for this group opting out of palliative care needs to be explored further.

Another predictor for opting out of care was receiving fewer visits from palliative care physicians. Physician visits are restricted to severe cases as charted out by the nurse. Therefore, we speculate that cases which received fewer visits from the physician are the ones which are less severe and therefore more likely to opt out of care.

Although existing evidence in literature states that males tend to use palliative care services lesser, we did not see this differentiation in this study [30]. Presence of a caregiver is said to predict utilization of palliative care. While some studies say that those who have unpaid caregivers tend to use palliative care less, others report that the use is enhanced when the patient has a care giver as they have someone to advocate on their behalf [43–46]. In our setting the presence of a caregiver especially spouse was not a predictor of continuing in palliative care.

One of the strengths of the study was that a large cohort of patients under palliative care was studied over a period of two decades.

There were also several limitations to this study. Firstly, we did not explore the quality of life of patients at enrolment and thereafter to quantify the impact of the programme on their lives. Secondly, the study did not explore the interim illnesses and immediate causes of death which would inform the specific professional skills needed for improving end of life care. Thirdly, the study also did not explore the reasons for opting out of home care. Fourthly, a long 20-year period of observation in this study might have seen changes in policy and practice related to palliative care. However, we have tried to take into account these changes to explain the patient enrolment and diagnosis pattern over the same period.

## Conclusion

This study has shown the changing dynamics of a palliative care programme in the Indian state of Kerala over a 20-year period, and highlights the need for similar programmes to remain flexible and able to adapt their services to a changing population in response to a growing global burden of NCDs. The variety of patients with different chronic conditions exemplifies the diverse range of clinical and psycho-social support skills required when caring for a cohort such as this one. The low rates of opting out of home care indicate the strengths of this care model. Future qualitative research is required to further understand the strengths and weaknesses of this home based care model and the experiences of the providers and caregivers.

## Abbreviations

CBO: Community Based Organisation
CVA: Cerebrovascular Accident
LSGIs: Local Self Government Institutions
NCDs: Non-Communicable Diseases
SMI: Serious mental illness
